# Improving Physical Activity, Athletic Performance, and School Climate in Disadvantaged Schools

**DOI:** 10.3390/children11121505

**Published:** 2024-12-10

**Authors:** Sigal Eilat-Adar, Michal Arnon, Nili Steinberg, Ronnie Lidor, Bosmat Sky

**Affiliations:** Levinsky-Wingate Academic College, Wingate Campus, Netanya 4290200, Israel; michalar@l-w.ac.il (M.A.); knopp@l-w.ac.il (N.S.); lidor@l-w.ac.il (R.L.); bosmat@l-w.ac.il (B.S.)

**Keywords:** intervention, physical activity, school climate, children, disadvantaged status

## Abstract

Background: Children are encouraged to spend 60 min each day performing moderate-to-vigorous physical activity. In this study, we assessed the impact of an intervention throughout the school year on physical activity, sports performance, and school climate in fifth–sixth-grade children from schools in a disadvantaged neighborhood. Methods: The intervention group (n = 44) participated in six weekly 45 min physical education classes; an athletic subgroup of these students participated in two additional weekly athletic classes. The control group (n = 73) participated in two standard weekly physical education classes. Pre- and post-intervention Eurofit Physical Fitness Tests were conducted. Results: Significant improvements were seen in the stand-and-reach test among girls in the intervention group [M = −0.47(7.71)–1.26(8.02) cm] compared to the control group [F(1,54) = 14.86, *p* < 0.01, η^2^ = 0.22]. No differences were seen between the groups in their daily physical activity, screen time, or school climate (*p* = 0.13, *p* = 0.17, and *p* = 0.35, respectively). Improvements were seen in the shuttle-run beep test, yet only in the athletic subgroup [F(1,93) = 60.38, *p* < 0.001, η^2^ = 0.39]. A trend towards significance for the largest improvement was seen in the athletic subgroup, who participated in eight weekly physical activity classes [F(2,93) = 3.75, *p* = 0.027, η^2^ = 0.07). Conclusions: Physical education curricula should enhance their focus on athletic performance, while increasing the number of weekly physical education classes in schools, to include daily lessons throughout the school week, each lasting at least 45 min.

## 1. Introduction

The World Health Organization (WHO) recommends that children aged 5–17 years spend at least 60 min each day performing moderate-to-vigorous (MV) physical activity (PA), with an emphasis on aerobic activities. Vigorous-intensity PA includes muscle and bone strengthening, which should be performed at least three times a week. Moreover, sedentary screen time should be limited, with recreational screen time not exceeding two hours per day [1]. Yet, despite the health benefits of PA, only a small number of children comply with these recommendations. In 2016, data were collected on 1.6 million children aged 11–17 years from 146 regions; the findings show that 81% of the participants were insufficiently physically active, with greater inactivity being seen in low socioeconomic populations [2].

Implementing PA in schools has short-term and long-term benefits, with as little as four-minute sessions being shown to improve behavior. Short PA sessions in the classroom have also been shown to positively affect attention and staying on task [3,4] and are a recommended tool for increasing the amount of daily PA among children [5]. In a meta-analysis of 115 studies on PA, covering almost 1.2 million young participants aged 9.1–17.7 years (with a high risk of bias), moderate PA (1–2 weekly hours) was found to have a smaller positive effect on academic performance than ≥3 weekly hours [6]. A narrative systemic review of 162 studies, covering more than 200,000 participants from 31 countries, showed a favorable effect for all patterns of PA (sporadic, bouts, and continuous) on the participants’ psychological and social well-being, cognitive health, bone health, weight, and indicators of adiposity [7]. Long-term benefits from PA, such as bone health cardiometabolic factors, were also observed [8]. Investing in the health and well-being of adolescents offers a “triple benefit”—improving their lives today, enhancing their future as adults, and positively impacting the next generation [9].

Since children spend 40% of their waking time at school [10], the school environment could have a meaningful impact on their PA levels. Early adolescence offers an important window of opportunity for impacting health behaviors [9] and well-being [11], especially given all that is known about psychological development during this stage [9]. In a narrative review of 21 studies, the factors that were found to most influence the implementation of health promotion within the school setting included organizational structure, educational program characteristics, fit with school policies and programs, and administrative support [12]. Yet, studies on health interventions in schools have led to conflicting results. Haerens et al. [13] suggested a pedagogical model for health-based physical education (HBPE), which was based on Jewett et al. [14]. One of the model’s central themes is “pupils valuing a physically active life”. Yet, to achieve this, the teachers’ beliefs must be oriented towards social reconstruction and self-actualization. The model also suggests that valuing physical activity is prominent in planning for learning. This is reflected in the local PE curriculum, whereby PE classes in schools “aim at developing, instilling, and nurturing PA and behavior patterns through teamwork, in line with the individual’s potential—to enhance their health, personality, and well-being” [15]. Moreover, when examining the impact of PA on school children, it is important to also address broader factors, including school climate. This term refers to the social atmosphere of the learning environment, in which students have different experiences, depending on a range of variables that are usually determined by teachers and administrators. Indeed, associations have been seen between a positive school climate and reduced emotional and behavioral issues at school, as well as increased academic achievements [16]. Yet studies are lacking on the impact of PA and school climate, especially when implementing health interventions and examining causal relationships.

The aim of this study, therefore, was to investigate the impact of a year-long PA intervention on the PA habits, sports performance, and school climate in elementary schools with a disadvantaged status. Our assumption was that both boys and girls could implement health-related changes acquired at school in their home lives.

## 2. Methods

### 2.1. Participants

At the onset of the study, 47 children from one school were assigned to the intervention group, and 84 children from a second school were assigned to the control group. The final data analysis was conducted for 44 participants (21 girls) from the intervention group (93.6%) and 73 participants (23 girls) from the control group (86.9%), who had completed all assessments and questionnaires. The participants from both groups were from grades 5–6, and both schools were located in the same disadvantaged neighborhood, as defined by the Ministry of Welfare. Additionally, 80% of the participants’ families were in touch with their local welfare department.

### 2.2. Procedures

The controlled intervention trial applied in this study was conducted between October 2021 and May 2022. The PE intervention was a part of a multidimensional health promotion program within the school setting, which was highly supported by the school administration and teachers. The school environment was adapted to enhance PA, educational programs were adapted to include nutritional activities, and the teachers were encouraged to participate in health activities in the teachers’ room. This article focuses on the PA aspect of the intervention.

The intervention was comprised of a number of PA-related components. First, the intervention group (n = 44) participated in six weekly PE classes. These took place Sunday to Friday, were conducted by the school’s PE teachers, and lasted 45 min each. Some participants from the intervention group (n = 20) also expressed their desire to participate in two additional 90 min athletic classes each week (i.e., the athletic subgroup). The aim of adding this subgroup was to assess the impact of higher cardiorespiratory exercises on aerobic performance. The control group (n = 73) participated in the standard twice-weekly PE classes, each lasting 45 min, with no additional activities being offered inside or outside the school.

All PE classes were conducted in line with the national PE curriculum [15]. In the school that participated in the intervention program, the playground was adorned with colorful floor markings, to encourage the children to play physical outdoor games (such as hopscotch). During each recess, the children were also given sports equipment to play with, such as balls, jump ropes, and frisbees. A 30 min active recess was held once a week, and a sports day was conducted once a month—organized by the teachers and the students. The teachers at this school were also taught how to implement PA into regular classes, in relation to the topics that they were teaching. Doing so offers children a short, yet active, break during lessons and, as such, can be beneficial [17]. Finally, the children were also encouraged to walk to and from school and to be active in the after-school hours as well.

### 2.3. Instrumentation

At the onset and completion of the intervention, the following athletic abilities, anthropometric measures, questionnaires, and school climate were assessed.

### 2.4. Athletic Achievements

To assess the participants’ physical ability, the following five Eurofit Physical Fitness Tests and anthropometric measures were applied: (1) single-leg balance test, 30 s (measured in numbers), for assessing balance; (2) stand-and-reach test (measured in cm), for assessing flexibility; (3) sit-up test, 30 s (measured in no. of repetitions), for assessing core strength; (4) 10 × 5 m shuttle run (measured in seconds), for assessing running speed and agility; and (5) 20 m endurance shuttle-run beep test (measured in seconds), for assessing aerobic cardiorespiratory endurance [18].

Anthropometric measurements included (1) height; (2) weight; and (3) fat percentiles, using the Tanita BC-418MA Segmental Body Composition Analyzer (Tanita Corporation, Tokyo, Japan) [19]. The body mass index (BMI) was then calculated. The WHO 2007 AnthroPlus software (Version 2019) was used to calculate the participants’ height-for-age, weight-for-age, and BMI-for-age z-scores (HAZ, WAZ, and BMIZ, respectively). Distributions of the z-scores were analyzed using descriptive and inferential statistical methods [20]. All measurements were conducted by highly experienced physical education teachers. Pre- and post-intervention tests were conducted by the same teachers.

### 2.5. Questionnaires

During a regular school lesson, the participants were also asked to complete a questionnaire on their demographic background, behaviors, and how they felt at school. Response options (scoring) were adapted to elementary school children and validated [21,22]. The questionnaires were distributed during regular school classes by a trained interviewer who explained to the children how to complete the questionnaire and remained with them while they were doing so.

### 2.6. School Climate

During a regular school lesson, the participants were also asked to complete a school-climate index questionnaire, comprised of 15 yes/no items. This tool was adopted from a questionnaire issued by the Ministry of Education [23], whereby the higher the school-climate score (0–15), the more positive the school climate. The questionnaire included yes/no items such as, “I like being at school” or “The teachers care about me.”

### 2.7. Data Analysis

Mean values (M) and standard deviations (SD) were calculated for continuous variables, while n (%) values were defined for categorical ones. Independent *t*-tests for pre- and post-intervention values were performed, to ensure that both groups (intervention and control) began the program with the same baseline. A two-way analysis of variance (ANOVA) with repeated measures was performed to test time, group, and time–group interactions, based on the Eurofit tests, the anthropometric measures, and the school climate index. A paired *t*-test was used to examine the pre-to-post-intervention change for each group. Tukey post hoc tests were conducted as a means for comparing between the three groups. Finally, when significant pre-intervention differences were seen between the groups, analysis of covariance (ANCOVA) was applied.

Anthropometric measures, PA, and fitness were examined for normality through skewness (SK < 2.0) and kurtosis (K < 7.00) procedures. Statistical analyses were performed using SPSS v.27.0 (IBM, Inc., Armonk, NY, USA), with a significance level for all tests set at α = 0.05.

### 2.8. Human Subject Approval Statement

The study was approved by the Ministry of Education (#11287) and by the Ethics Committee at the authors’ affiliated academic institution (#267). Additionally, the parents of all participating children submitted signed informed consent forms.

## 3. Results

### 3.1. Country of Origin

Few participants in the intervention group were first-generation immigrants compared to about one-third in the control group (4.7% and 34.2%, respectively, *p* < 0.01). Almost all participants (in both groups) were second-generation immigrants. In the intervention group, 86.4% of the parents (one or both) were immigrants, mainly from developing countries (72.7%). In the control group, on the other hand, 87.7% of the parents (one or both) were immigrants, with about half having immigrated from developing countries (50.7%, *p* < 0.03).

### 3.2. Anthropometric Comparisons

As the anthropometric and PA measurements were normally distributed (except for the balance test), these were analyzed as continuous variables. When comparing each group pre- and post-intervention, a significant reduction was seen in the BMI percentiles of the male participants in both groups, with an interaction between groups [F(1,51) = 7.69, *p* < 0.001, η^2^ = 0.131]—indicating that a larger reduction in BMI was seen in the intervention group. No significant differences were found in the z-scores (see Table 1 and Table 2). A significant reduction was also seen in the BMI percentiles of the female participants in both groups [F(1,59) = 38.24, *p* < 0.01, η^2^ = 0.33], with decreased z-scores in both groups and with an interaction [F(1,59) = 4.21, *p* = 0.05, η^2^ = 0.07] and within normal z-score ranges (see Table 1 and Table 2).

### 3.3. PA Measurements

In the male participants, the Eurofit test results revealed a significant time effect in all parameters (*p* < 0.05), except for the stand-and-reach test, where reduced results were seen in both groups following the intervention. In the sit-up test, a significant time effect (*p* < 0.01) and a significant time–group interaction were found [F(1,45) = 6.68, *p* < 0.05, η^2^ = 0.129]. Yet in the control group, the difference was conveyed solely through two additional repetitions in the test, which was not clinically significant. Finally, in the control group, the Chi^2^ test showed a significant time effect in the balance test (*p* = 0.01). No other interactions were observed in any other measurements (see Table 1).

In the female participants, a significant time–group interaction was revealed between groups in the stand-and-reach flexibility test, with an increase demonstrated in the intervention group and a decrease in the control group [F(1,54) = 14.86, *p* = 0.01, η^2^ = 0.22]. However, for the sit-up test [F(1,54) = 3.93, *p* = 0.05, η^2^ = 0.067] and the beep test [F(1,50) = 5.84, *p* = 0.02, η^2^ = 0.105], a larger improvement was seen in the control group. No changes or differences between the groups were observed in any other fitness tests (see Table 2).

### 3.4. Athletic Subgroup

**Figure 1:**
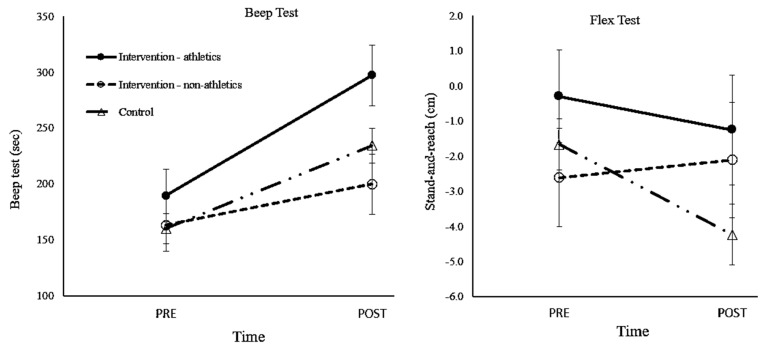
Beep test (endurance) and stand-and-reach (flexibility) results by subgroup.

Sensitivity analysis was conducted to compare between the athletic subgroup (n = 20), the non-athletic intervention subgroup (n = 21), and the control group (n = 73). No significant differences were seen during the pre-intervention analysis (except for the sit-up test). A mixed repeated measures ANOVA (pre- and post-intervention timepoints X three groups) was conducted for each dependent variable. The results for the beep test (cardiorespiratory endurance) revealed a significant time effect [F(1,93) = 60.38, *p* < 0.001, η^2^ = 0.39], a non-significant group effect [F(2,93) = 60.38, *p* = 0.148, η^2^ = 0.040], and a trend towards a significant time–group interaction [F(2,93) = 3.75, *p* = 0.027, η^2^ = 0.07]. These findings indicate that the changes seen in the participants over time differed between the three groups. The athletic subgroup showed greater improvement in the beep test compared to their counterparts (Figure 1), as follows: athletic subgroup: pre- 189.58 ± 96.69 vs. post-intervention 297.11 ± 115.80 sec, *p* < 0.001; non-athletic intervention subgroup: pre- 163.37 ± 66.05 vs. post-intervention: 199.74 ± 113.17 sec, *p* = 0.05; and control group: pre- 160.31 ± 113.35 vs. post-intervention 234.38 ± 120.49 sec, *p* < 0.001.

A mixed repeated measures ANOVA for flexibility revealed a significant time–group interaction [F(2,100) = 3.10, *p* = 0.05, η^2^ = 0.06], indicating differences in changes over time between the three subgroups. No significant change was observed in flexibility in the athletic subgroup (pre- −0.30 ± 7.39, post-intervention −1.25 ± 8.26, *p* = 0.31) or in the intervention non-athletic group (pre- −2.61 ± 6.55 vs. post-intervention −2.11 ± 8.34, *p* = 0.75). However, a significantly reduction in flexibility was seen in the control group (pre- −1.67 ± 5.19 vs. post-intervention −4.23 ± 6.12, *p* < 0.001).

A covariate analysis revealed a larger improvement in the sit-up test in the control group than in the athletic group. No time–group interactions were seen between the athletic subgroup and the other two groups in the shuttle run or the single-leg balance tests. In summary, the athletic subgroup showed improved beep-test results and less reduction in flexibility than the other groups.

### 3.5. Daily PA and Screen Time

As seen in Table 3, no differences were found in the daily PA and screen time between the intervention and control groups.

### 3.6. School Climate

**Figure 2:**
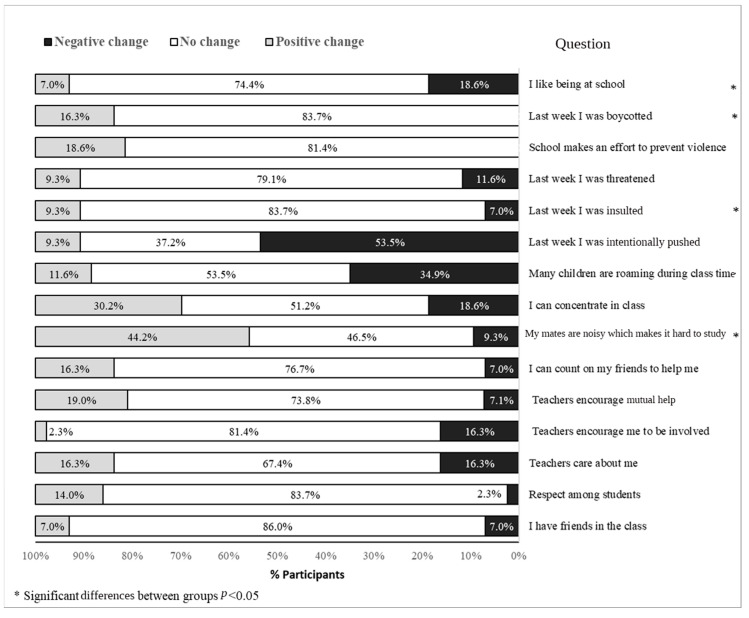
Pre-to-post school climate in the intervention group (n = 44).

**Figure 3:**
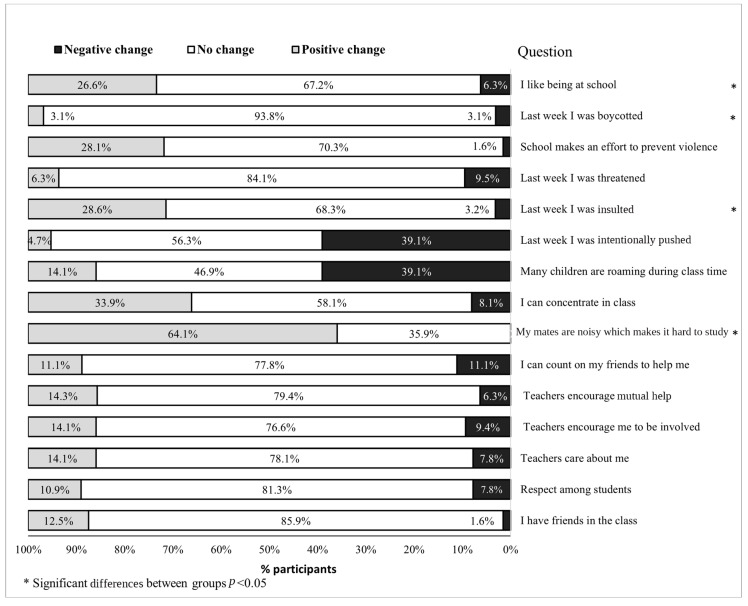
Pre-to-post school climate in the control group (n = 70).

No significant differences were seen between the intervention group and the control group in the average school climate index: pre-intervention, 10.86 ± 0.44 vs. 10.48 ± 0.34, respectively, and post-intervention 10.93 ± 0.55 vs. 11.32 ± 0.43, respectively. No differences were seen between times (*p* = 0.27), and no interaction was observed (*p* = 0.35). The trends in the change in school climate, from the pre- to the post-intervention (increased, no change, or decreased) are presented for the intervention group in Figure 2 and for the control group in Figure 3 for all 15 items of the school climate index.

## 4. Discussion

This study aimed at examining the impact of an intervention program that was conducted throughout most of the school year on PA, athletic achievements, screen time, and school climate. The participants included fifth- and sixth-grade children from two schools in a disadvantaged neighborhood. In general, the results showed that only an intensive PA intervention succeeded in improving endurance.

### 4.1. Eutofit Test

A significant time effect, with improved fitness measurements was seen in males, with an improvement in most parameters in females. This indicates an impact of biological maturity (i.e., age) [24]. However, both genders in both groups exhibited improved beep-test results, which may be also explained by growth at that age. Notably, gender differences are not the aim of the current study since girls tend to mature about two years earlier than boys, which lowers their lean peak bone mass [24]; additionally, as boys are more engaged in MVPA [25], this may explain the differences between genders, as may cultural and preferential differences. For example, the significant time effect seen in males in both groups can be explained by age and growth. A decreased flexibility was seen in male participants in both groups. This finding could stem from observations that boys are more likely than girls to select contact, competition, and team activities (such as ball games), which may reduce flexibility [25]. Moreover, flexibility in boys, in general, has been seen to decrease over time [26]. The lack of time effect of the shuttle-run, stand-and-reach, and balance tests in females is a result of opposite changes in the intervention and control female groups.

Despite the large increase in weekly PA classes in the intervention group, no clear differences were seen between the groups when assessing the Eurofit test results. The control group exhibited improved sit-up test results. In the stand-and-reach flexibility test, the female participants in the intervention group demonstrated improvement, while their female counterparts in the control group presented decreased flexibility.

Since girls are more likely to select individual and non-contact activities that incorporate artistic exercises (such as gymnastics, dance, and aerobic dance), which increase flexibility [27], daily PA classes increased flexibility exercises among girls in the intervention group. Future studies could also benefit from examining the physical activities in which the participants take part outside of school hours.

The lack of improvement in most fitness skills in this study, despite the intervention program, may have stemmed from insufficient vigorous PA during the daily PE classes. These findings are in line with previous studies that did not find a favorable effect of PA interventions on improved PA in children. Da Costa et al. found that vigorous PA only comprised about 16% of a PE class [28]. During the last few years, studies have aimed at understanding how to increase the MPVA in PE classes. In a systematic review of the factors correlated with MVPA during PE classes in elementary school, there were 42 studies with an MVPA outdoor class, a PE context (fitness, game play, and skill practice), perceived competence, and smaller classes [25]. Although all these factors existed in the current intervention, it is possible that the Eurofit battery of tests used in the current study was not sensitive enough to detect such small differences, despite it being considered an acceptable means for measuring changes in fitness abilities [18].

### 4.2. Athletic Performance Goals in PA Classes

The PE classes did not specifically aim at improving athletic performance but rather emphasized playing games and having fun—as a means for increasing daily PA. It is therefore important to address the positive changes seen in the participants of the athletic subgroup—specifically in the shuttle-run beep test and in the stand-and-reach flexibility test, which highlights the importance of setting clear goals (such as endurance training) and more practice time. Future studies should define more specific goals and instructions in their PA intervention programs.

### 4.3. PA Time, School Climate, and Screen Time

No changes in the participants’ PA time, school climate, and screen time were observed. A review of 20 randomized controlled school-based interventions was conducted, with all interventions lasting at least 12 weeks and in young participants aged 6–18 years. Significant improvements were mainly observed in the children’s school-related PA rather than in their leisure-time PA [28]. Similar results were shown in a Cochrane review of 26 studies [29]. In a later analysis of 73 countries, three PE classes and above per week were significantly associated with lower leisure time PA in both the middle east region and low-income subgroup [30].

### 4.4. Parents’ Role

Another explanation for this lack of change could be attributed to the crucial role that parents and the home environment play in the lifestyles of children [31,32]. Without family members’ involvement, it is unlikely that children will improve their PA time and level [33]. Our assumption that children will implement health-related changes that they acquire at school in their home life may have failed, due to cultural and language gaps between the school and the home environments in this study, as seen in previous research [34]. To ensure parental involvement in future health-related programs, it is important to incorporate cultural characteristics, such as fitting in with their dominant language [35] and possibly also with their PA preferences. While the intervention was adapted to local norms, most parents in the intervention group were born in developing countries, where leisure-time PA is less common [36]. Future research could benefit from adapting the PA after school to cultural norms at home, while involving the parents in the process—such as conducting interesting and inviting talks with the parents, where the importance of PA is conveyed. An additional difficulty in implementing this health-related program at home could stem from the parents’ limited financial resources and long work hours—as the study took place in a disadvantaged neighborhood. Such circumstances often limit parents’ options for providing PA-related modeling [37].

### 4.5. Baseline PA Engagement

At the baseline, more than half the participants in the intervention group and almost half in the control group reporting being engaged in at least 60 min of daily PA. These numbers are relatively high. In an analysis of 298 school-based surveys from 146 countries and regions, only 19% of children achieved daily PA recommendations [2]. As such, this relatively high PA engagement rate at the onset of the study could explain the lack of change in PA in either group following the intervention. Alternatively, children in the intervention group may have increased their PA time at school and paradoxically decreased their leisure-time PA without reporting it.

### 4.6. School Climate

No significant time effect was seen in the mean school climate index. This finding could be explained by the school climate being multifaceted [38]. It is possible that a school with a positive climate may lead to enhanced PA—yet, not vice versa. This may also explain the lack of difference in PA involvement between the two groups.

### 4.7. Implications for School Health Policy, Practice, and Equity

These results suggest that schools should integrate more MVPA—while striving to enhance athletic performance and offer their students an enticing setting that encourages staying active. In addition, assessment tools should be adapted to the goals of the specific PE curriculum.

### 4.8. Limitations and Future Research

The controlled intervention program presented in this study was carefully planned in both detail and structure. Moreover, the intervention was implemented for most of the school year. As such, this novel study has important practical and theoretical implications. However, several research limitations should be addressed. First, a significant difference was seen between the two groups in the country of origin of the participants’ parents. This may have impacted the parental modeling to which the young participants were exposed, including health behaviors and leisure habits. A second limitation that should be addressed is the timing of the study, which was conducted immediately following the COVID-19 era. At this time, although schools were open, wearing a face mask was obligatory and may therefore have caused discomfort among the children and led to reduced activity. However, this is not expected to be a differential bias between the groups. A longer intervention program or a follow-up study could decrease the possible impact of such limitations.

Additionally, the study did not track or measure the different types of PA that were applied during the lessons, nor did it examine the degree of physical exertion required. As such, different levels of PA may have led to different outcomes. Moreover, the internal validity of the Eutofit tests was not measured. Thereby, these tests may not have been adequately in line with the PA classes, activities, or curricula. Future intervention programs could benefit from specifying the PA that should be incorporated in the daily lessons, and the assessment tools should be selected accordingly.

Finally, the self-reporting questionnaires may have been impacted by both recall and agreement bias, especially as the young participants were asked to complete the questionnaires during class. However, should such bias exist, it is not expected to be differential between the two groups. Future studies could benefit from tracking the participants’ physical activity through new technologies—as a means for achieving more objective and accurate data regarding their PA during their private leisure time.

## 5. Conclusions

Improved athletic endurance in boys and flexibility in girls were seen following daily PE lessons in a PA-enhanced school setting. However, improved beep test results were only seen in the group that participated in six weekly PE classes and two additional weekly athletic training classes. As such, there is a need for more than six weekly PE classes in schools, in order to improve athletic performance—with at least two of these classes focusing on athletic performance. Alternatively, a specific emphasis should be placed on athletic skills during PA lessons in schools, emphasizing self-improvement rather than gold-standard achievements. Finally, future studies should provide parents with a cultural adjustment program, to increase their involvement, examine longer intervention periods, and conduct follow-up assessments.

## Figures and Tables

**Table 1:** Anthropometric and PA measures in the male participants.

Measurement	Intervention (n = 22)			Control (n = 39)					
Mean (SD)	Pre-Intervention	Post-Intervention	*p* Value Pre-Post	Pre-Intervention	Post-Intervention	*p* Value Pre-Post	*p* Value Between Times	*p* Value Between Groups	*p* Value Interaction
Weight (kg)	35.6(12.5)	36.2(12.4)	0.11	43.1(11.5)	44.6(11.3)	<0.01 *	<0.01 *	0.02 *	0.15
Height (m)	1.4(0.1)	1.5(0.1)	<0.01 *	1.5(0.1)	1.5(0.1)	<0.01 *	<0.01 *	0.23	<0.01 *
BMI percentile (%)	46.2(36.7)	2.6(33.2)	<0.01 *	73.3(28.8)	64.4(33.2)	<0.01 *	<0.01 *	<0.01 *	<0.01 *
Beep test (s)	196.1(83.2)	321.0(98.0)	<0.01 *	208.63(142.1)	283.4(138.0)	<0.01 *	<0.01 *	0.72	0.07
Stand-and-reach test (cm)	−2.3(6.3)	−4.6(7.5)	0.08	−2.3(3.5)	−3.9(5.5)	0.07	<0.01 *	0.81	0.67
Sit-up test (no.)	18.7(4.3)	21.2(5.9)	0.01 *	19.5(5.0)	24.8(4.9)	<0.01 *	<0.01 *	0.13	0.01 *
Shuttle test (s)	15.3(1.0)	15.7(1.2)	0.08	14.8(1.5)	15.0(1.4)	0.403	0.04 *	0.16	0.22
Balance test no. of falls in 30 s (%)	13/18(0.7)	16/18(0.9)	0.21	18/30(0.6)	27/30(0.9)	0.01 *	NA **		

* Significant difference *p* < 0.05, ** Not applicable.

**Table 2:** Anthropometric and PA measures in the female participants.

Measurement	Intervention (n = 19)			Control (n = 40)					
Mean (SD)	Pre-Intervention	Post-Intervention	*p* Value Pre-Post	Pre-Intervention	Post-Intervention	*p* Value Pre-Post	*p* Value Between Times	*p* Value Between Groups	*p* Value Interaction
Weight (kg)	42.6(11.0)	44.6(10.7)	<0.01 *	44.1(11.5)	46.4(12.4)	0.01 *	<0.01 *	0.60	0.85
Height (m)	1.5(0.1)	1.5(0.1)	<0.01 *	1.5(0.1)	1.5(0.1)	<0.01 *	<0.01 *	0.78	<0.01 *
BMI percentile (%)	66.0(32.2)	54.3(35.6)	<0.01 *	71.0(30.5)	63.9(31.9)	<0.01 *	<0.01 *	0.39	0.14
Beep test (s)	160.6(80.8)	188.9(110.0)	0.04 *	118.6(51.0)	185.2(86.3)	0.03 *	<0.01 *	0.36	0.02 *
Stand-and-reach test (cm)	−0.47(7.7)	1.3(8.0)	0.12	−1.2(6.0)	−4.5(6.6)	<0.01 *	0.25	0.08	<0.01*
Sit-up test (no.)	15.6(6.5)	16.6(5.0)	0.41	12.9(3.5)	16.5(5.6)	<0.01 *	<0.01 *	0.29	0.05 *
Shuttle test (s)	16.8(2.6)	17.1(2.0)	0.42	19.1(5.2)	16.0(1.3)	0.27	0.38	0.06	0.43
Balance test no. of falls in 30 s (%)	15/21(71.4)	17/21(81.0)	0.47	25/38(65.8)	30/38(78.9)	0.20	NA **		

* Significant difference *p* < 0.05, ** Not applicable.

**Table 3:** PA and screen time.

Variable	Participants’ Response	Intervention Group		Control Group			
		Pre-Intervention n (%)	Post-Intervention n (%)	Pre-Control n (%)	Post-Control n (%)	Pre- Between Groups (*p* Value)	Post- Between Groups (*p* Value)
Perform PA for at least 60 min	0–1 times per week	7(15.9)	6(14.3)	13(17.8)	8(12.1)	0.16	0.13
	2–4 times per week	13(29.5)	11(26.2)	33(45.2)	30(45.5)		
	5–7 times per week	24(54.5)	25(59.5)	27(37.0)	28(42.4)		
Daily screen time	≤60 min per day	13(29.5)	11(25.6)	17(23.3)	19(23.5)	0.75	0.17
	2–3 h per day	16(36.4)	13(30.2)	29(39.7)	32(47.1)		
	≥4 h per day	15(34.1)	19(44.2)	27(27.0)	20(29.4)		
	Important/very important	32(72.7)	25(65.8)	56(80.0)	59(89.4)		

## Data Availability

The data presented in this study are available on request from the corresponding author. The data are not publicly available due to privacy and ethic.
